# Next-Generation CAR-T and TCR-T Cell Therapies for Solid Tumors: Innovations, Challenges, and Global Development Trends

**DOI:** 10.3390/cancers17121945

**Published:** 2025-06-11

**Authors:** Tomomi Sanomachi, Yuki Katsuya, Tetsuya Nakatsura, Takafumi Koyama

**Affiliations:** 1 Department of Experimental Therapeutics, National Cancer Center Hospital, Tokyo 104-0045, Japan; 2 Division of Cancer Immunotherapy, Exploratory Oncology Research and Clinical Trial Center, National Cancer Center, Kashiwa 277-8577, Japan

**Keywords:** CAR-T cell therapy, TCR-T cell therapy, adoptive cell therapy, solid tumors, intellectual property, patent landscape

## Abstract

Chimeric antigen receptor (CAR)-T and T-cell receptor (TCR)-engineered T-cell (TCR-T) therapies have revolutionized the treatment of hematological malignancies; however, their application to solid tumors remains a formidable challenge. The immunosuppressive tumor microenvironment, antigen heterogeneity, and manufacturing complexity limit the clinical efficacy and scalability of these treatment modalities. This review provides a comprehensive analysis of the current clinical development strategies for CAR-T and TCR-T cell therapies for solid tumors. Herein, we discuss recent breakthroughs and highlight the potential of TCR-T cell therapy. Furthermore, innovative approaches for enhancing CAR-T cell function in solid tumors (e.g., in vivo engineering; induced pluripotent stem cell-derived allogeneic CAR-T cells; armored CAR constructs; dual-antigen targeting; and combination regimens with checkpoint inhibitors, chemotherapy, radiotherapy, and oncolytic viruses) are explored. We also present trends in global patent activity, revealing a marked acceleration in CAR-T- and TCR-T-related innovations, with the United States and China leading with respect to application volumes. This field is increasingly characterized by multidisciplinary collaborations between academia and industry, driving the development of next-generation platforms, including messenger RNA-based and off-the-shelf cell therapies. Although no CAR-T product has been approved for solid tumors, these findings underscore the accelerating momentum and translational promise of adoptive cell therapies. Addressing the unique biological and logistical challenges of solid tumors is essential for realizing the full potential of these transformative immunotherapies.

## Simple Summary

Chimeric antigen receptor (CAR)-T and T-cell receptor (TCR)-engineered T-cell (TCR-T) therapies application to solid tumors remains a formidable challenge. This review provides a comprehensive analysis of the current clinical development strategies for CAR-T and TCR-T cell therapies for solid tumors. The authors aim to explore recent strategies that may overcome barriers like the complex tumor environment, differences in target proteins, and difficulties in producing these therapies. This review also highlights new ideas being tested, such as directly modifying T cells inside the body or combining cell therapies with other treatments. The study shows that global research and patents in this field are rapidly growing, especially in the United States and China. By identifying key innovations and challenges, this review may help researchers and companies develop more effective, widely available immune cell therapies for patients with solid tumors.

## 1. Introduction

Chimeric antigen receptor (CAR)-T and T-cell receptor (TCR)-engineered T-cell (TCR-T) therapies have revolutionized the treatment of hematological malignancies; however, their application to solid tumors remains a formidable challenge. The immunosuppressive tumor microenvironment, antigen heterogeneity, and manufacturing complexity limit the clinical efficacy and scalability of these treatment modalities. This review provides a comprehensive analysis of the current clinical development strategies for CAR-T and TCR-T cell therapies for solid tumors.

## 2. Overview of CAR-T Cell Therapy for Cancer

CAR-T cell therapy is an innovative immunotherapeutic modality in which autologous T cells are genetically engineered to target and eliminate cancer cells. This approach has demonstrated remarkable clinical efficacy, particularly for hematologic malignancies [1,2] and has garnered significant global attention. In Japan, CAR-T cell therapies for several indications, including relapsed/refractory CD19-positive B-cell acute lymphoblastic leukemia in patients aged ≤25 years, large B-cell lymphoma, follicular lymphoma, and multiple myeloma, have been reimbursed under the national health insurance system.

**Figure 1:**
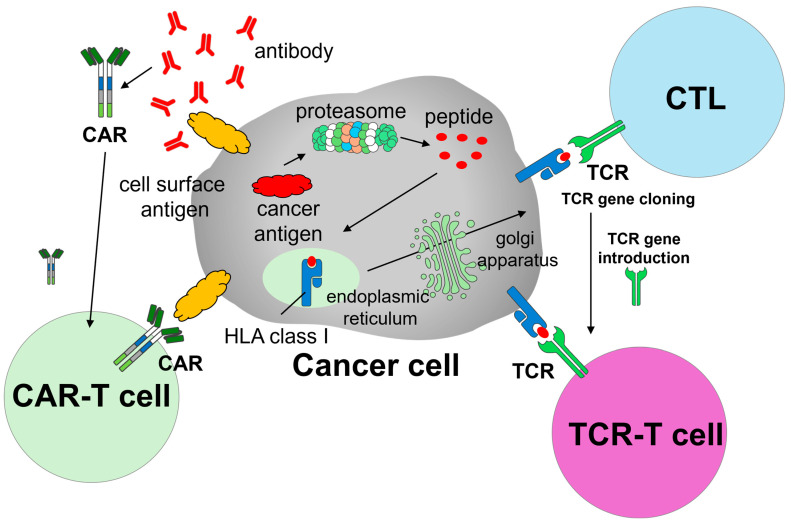
Schematic illustration of CAR-T and TCR-T cell therapies. This figure presents a conceptual diagram of CAR-T cell therapy and TCR-T cell therapy. Abbreviations: CAR, chimeric antigen receptor; CAR-T, chimeric antigen receptor T-cell; CTL, Cytotoxic T Lymphocyte; HLA, human leukocyte antigen; TCR, T-cell receptor; TCR-T, T-cell receptor-engineered T-cell.

CAR-T cells are produced by introducing chimeric antigen receptors (i.e., CARs) into autologous T cells. CARs are designed to recognize tumor-associated surface antigens, enabling engineered T cells to specifically identify and eliminate malignant cells (Figure 1). The manufacturing process consists of multiple stages: leukapheresis, transportation to a Good Manufacturing Practice (GMP)-compliant manufacturing facility, viral transduction or other gene editing to insert the CAR constructs, ex vivo expansion, and rigorous quality control, followed by reinfusion into the patient. Prior to infusion, patients typically undergo bridging therapy and lymphodepleting chemotherapy to enhance CAR-T cell engraftment and efficacy.

Although CAR-T cell therapies have yielded high response rates in hematological cancers, their clinical benefits in solid tumors remain limited. Major barriers include the immunosuppressive tumor microenvironment (TME); heterogeneous or low antigen expression; antigen loss; poor tumor infiltration; and T cell exhaustion driven by the persistent stimulation of inhibitory receptors, such as PD-1 and LAG-3 [3]. Various strategies have been explored to overcome these limitations, including optimizing antigen selection, improving CAR design, incorporating resistance mechanisms against TME-induced suppression, and enhancing T cell trafficking to tumor sites. However, serious adverse events should be carefully managed. Prominent toxicities include cytokine release syndrome [4,5], immune effector cell-associated neurotoxicity syndrome [6], and hemophagocytic lymphohistiocytosis-like syndrome [7]. These complications could be life-threatening and require vigilant monitoring.

Unlike for hematologic malignancies, CAR-T cell therapy for solid tumors faces challenges unique to the tissue context. For example, on-target/off-tumor toxicity is more common in normal tissues owing to the expression of target antigens, such as human epidermal growth factor receptor 2 and mesothelin [3]. Dense extracellular matrices may also restrict CAR-T cell infiltration and migration. Moreover, the TME in solid tumors is enriched with immunosuppressive components, such as regulatory T cells, M2 macrophages, myeloid-derived suppressor cells, and cytokines (e.g., transforming growth factor-β and interleukin [IL]-10). This hostile environment diminishes CAR-T cell activity and efficacy. Finally, the typically high tumor burden in solid tumors can lead to chronic antigen stimulation, resulting in T cell exhaustion and diminished antitumor responses. These complex barriers underscore the pressing need for next-generation CAR-T cell therapies specifically engineered to function effectively in solid tumors.

## 3. Overview of TCR- T Cell Therapy for Cancer

TCR-T cell therapy represents another promising adoptive cell therapy strategy, in which autologous T cells are genetically modified to express tumor-specific TCRs. Unlike CAR-T cells, which target surface antigens in a major histocompatibility complex (MHC)-independent manner, TCR-T cells recognize intracellular tumor-derived peptides presented by MHC molecules, enabling them to target a broader range of tumor-associated antigens [8,9,10] (Figure 1). This MHC-dependent mechanism enables TCR-T cells to recognize intracellular tumor-associated or tumor-specific antigens that are processed and presented via MHC molecules, such as cancer/testis antigens (e.g., NY-ESO-1 and MAGE-A4) and neoantigens derived from somatic mutations (e.g., KRAS G12D) [11]. This expands the range of targetable tumor antigens beyond cell surface molecules, which are the sole targets of CAR-T cells. The overall manufacturing process for TCR-T cells is similar to that for CAR-T cells: T cells are collected from the patient, transduced or edited to express tumor-specific TCRs, expanded ex vivo under GMP conditions, and reinfused following lymphodepletion. Table 1 outlines the key differences between CAR-T and TCR-T cell therapies.

In August 2024, the United States (US) Food and Drug Administration (FDA) granted accelerated approval for afamitresgene autoleucel, a TCR-T cell therapy targeting the MAGE-A4 antigen presented by a specific human leukocyte antigen (HLA) type, for unresectable or metastatic synovial sarcoma. This approval was based on the SPEARHEAD-1 trial, which demonstrated an overall response rate of 39% among 44 patients [12]. This marked a milestone for TCR-T cell therapy in solid tumors and initiated further clinical development across a range of malignancies. However, despite its potential, TCR-T cell therapy faces several challenges. Many of these issues overlap with those encountered by CAR-T cells, such as limited tumor infiltration owing to physical barriers, immunosuppressive TMEs, T cell exhaustion, and the risk of on-target/off-tumor toxicity when targeting shared antigens. However, TCR-T cells rely on MHC expression; therefore, their therapeutic efficacy may be impaired by tumor-driven MHC down-regulation, a common immune evasion mechanism in advanced cancers.

Moreover, the HLA system is highly polymorphic, and each TCR construct must be tailored to a specific HLA type, posing a significant challenge for broad clinical application [9]. These factors highlight the need for individualized approaches to TCR-T cell therapy and represent key bottlenecks in its development and scalability. However, ongoing research to overcome these limitations, including improvements in TCR affinity, persistence, and safety, continues to drive progress toward optimized TCR-T cell therapies for solid tumors.

## 4. Strategies to Enhance the Efficacy of CAR-T Cell Therapy Against Refractory Solid Tumors

### 4.1. CAR-T Cell Approval Strategy for Solid Tumor Treatment

There has been notable progress in applying TCR-T cell therapy for refractory solid tumors, with one product, afamitresgene autoleucel, receiving FDA approval for unresectable or metastatic synovial sarcoma. However, CAR-T cells have not been approved for solid tumors. The major barriers include tumor antigen escape, lack of identifying effective tumor-specific surface antigens, the immunosuppressive tumor microenvironment, and difficulties in T cell infiltration [13,14,15]. Overcoming these challenges is crucial to achieving success in CAR-T cell therapy for solid tumors. Although numerous clinical trials are ongoing worldwide, this review does not attempt to summarize them comprehensively. Instead, we recommend recent high-quality reviews focusing on CAR-T cell combination strategies, such as those by Uslu et al. [16].

### 4.2. In Vivo CAR-T Cell Therapy

Gene therapies can be broadly categorized into ex vivo and in vivo modalities (Table 2). Currently approved CAR-T cell therapies for hematologic malignancies are ex vivo gene therapies that require leukapheresis, T-cell modification and expansion, and reinfusion. This process demands significant infrastructure, personnel, and financial resources and limits accessibility. In contrast, in vivo gene therapy involves the direct delivery of gene-editing components (e.g., viral or non-viral vectors) to patients, potentially bypassing the need for leukapheresis and complex manufacturing [17]. Efforts to generate CAR-T cells in vivo have demonstrated a proof-of-concept in murine tumor models using injectable viral vectors or nanoparticle platforms [18]. However, although these approaches are promising, the long-term safety concerns remain unresolved. Potential risks include persistent antigen stimulation, unexpected recognition of normal cells, off-target effects due to nonspecific interactions, and unintended immune activation. Integrating vectors, such as lentiviruses, may result in insertional mutagenesis [19], and individual differences in immune cell composition and thymic selection add further uncertainty. To mitigate these risks, research is shifting toward non-integrating vector systems (e.g., adeno-associated virus [AAV] vectors [18,20]) or transient gene expression platforms (e.g., messenger RNA [mRNA] delivery via lipid nanoparticles [LNPs]) [21,22]. These strategies aim to enable short-term CAR expression while reducing genotoxic risk.

### 4.3. Allogeneic iPSC-Derived CAR-T Cell Therapy

Allogeneic CAR-T cell therapy, especially induced pluripotent stem cell (iPSC)-derived CAR-T cells, is gaining attention as a scalable off-the-shelf option. These therapies use T cells derived from healthy donors, modified to inactivate TCRs (to reduce graft-versus-host disease [GVHD] risk), and stored as ready-to-use frozen products. Although this approach circumvents the logistical challenges of autologous therapy and reduces production time and cost, significant concerns regarding long-term persistence and immune rejection, including long-term GVHD over the long term, remain [23].

### 4.4. Developing Strategies for CAR-T Cell Therapy

Several approaches that can be applied to both autologous and allogeneic CAR-T cell platforms have been developed. Armored CAR-T cells or T cells redirected for universal cytokine-mediated killing are engineered to secrete cytokines [24] or express costimulatory ligands (e.g., CD28L and 4-1BBL), enhancing survival in the hostile TME. These cytokines can also recruit innate immune cells, such as natural killer (NK) cells and macrophages, amplifying antitumor responses. Dual- or multitargeting-CAR-T cells are currently being developed to address antigen escape. For example, relapse following anti-CD19 CAR-T cell therapy occurs in 30–60% of cases [25], and is one of the main reasons associated with CD19 loss. To overcome this problem, dual-targeting CAR-T cells (e.g., CD19/CD22) and multiple-targeting CARs are under investigation [26].

Combination therapies are also being investigated. These include immune checkpoint inhibitors (e.g., anti–PD-1, anti–CTLA-4) that restore T cell activity by blocking inhibitory pathways; chemotherapy and radiotherapy that may enhance CAR-T cell infiltration and promote immunogenic tumor cell death; molecularly targeted therapies that modulate tumor signaling pathways; and other immunotherapies, such as cancer vaccines and onco-lytic viruses. However, although combination approaches hold promise, they introduce new challenges, such as optimal sequencing, dosing strategies, and cost-effectiveness.

### 4.5. Enhancing Long-Term Safety

Long-term safety is an increasingly important concern for CAR-T cell therapy. In 2023, the FDA reported 22 cases of secondary T-cell cancers in more than 27,000 CAR-T dose [27]. Although causality remains uncertain, two main considerations are worth noting: (1) secondary malignancies can also arise from prior therapies, including chemotherapy, radiotherapy, and stem cell transplantation, with reported prevalence rates of one in six patients [28], and (2) current data suggest that T-cell lymphoma post-CAR-T cell therapy occurs in 0.22% of cases [29]. Although further research is required to clarify whether these cases are causally associated with CAR-T cell therapy, they have raised concerns regarding integration-based CAR technologies. Transient expression strategies using AAV vectors or mRNA, or LNPs may offer safer alternatives.

### 4.6. Beyond T Cells: CAR-Modified Myeloid and Innate Immune Cells

Although T cells remain the cornerstone of adoptive cell therapy, other immune cell types, such as macrophages, neutrophils, and NK cells, are being investigated for CAR engineering. These cells lack TCRs and therefore carry a lower risk of side effects. However, their use is limited by poor tumor tropism, susceptibility to TME-mediated suppression, functional exhaustion.

To address these issues, emerging strategies include the introduction of tumor-homing receptors (e.g., CCR2 and CXCR2); optimization of intracellular signaling domains; synthetic receptors (e.g., PD-1-CD28); cytokine co-expression (e.g., IL-7, IL-12, IL-15, and IL-18); use of rejuvenated or stem-like immune cells (e.g., iPSC-derived or stem cell-like memory T cells); and bispecific or dual-antigen recognition systems. Such developments may enhance the applicability and efficacy of cellular immunotherapies.

## 5. Global Development of CAR-T and TCR-T Cell Therapies for Solid Tumors: An Intellectual Property Perspective

### 5.1. Trends in Patent Filings

Building upon the preceding sections outlining CAR-T and TCR-T cell therapies, this section provides an advanced analysis of global development trends in these therapies for solid tumors based on a comprehensive patent landscape analysis conducted in collaboration with Clarivate (London, United Kingdom [UK], and Philadelphia, PA, US). Patent data were retrieved from the Derwent Innovation-DWPI and Cortellis Competitive Intelligence (CCI)-Patents databases, covering a broad range of intellectual properties related to CAR-T and TCR-T technologies. Between 2003 and 2011, the number of patent filings remained modest at approximately 50 per year, but it started to increase around 2012. This upward trend continues, reflecting a growing global interest in adoptive cell therapies.

### 5.2. Leading Countries and Filing Strategies

**Figure 2:**
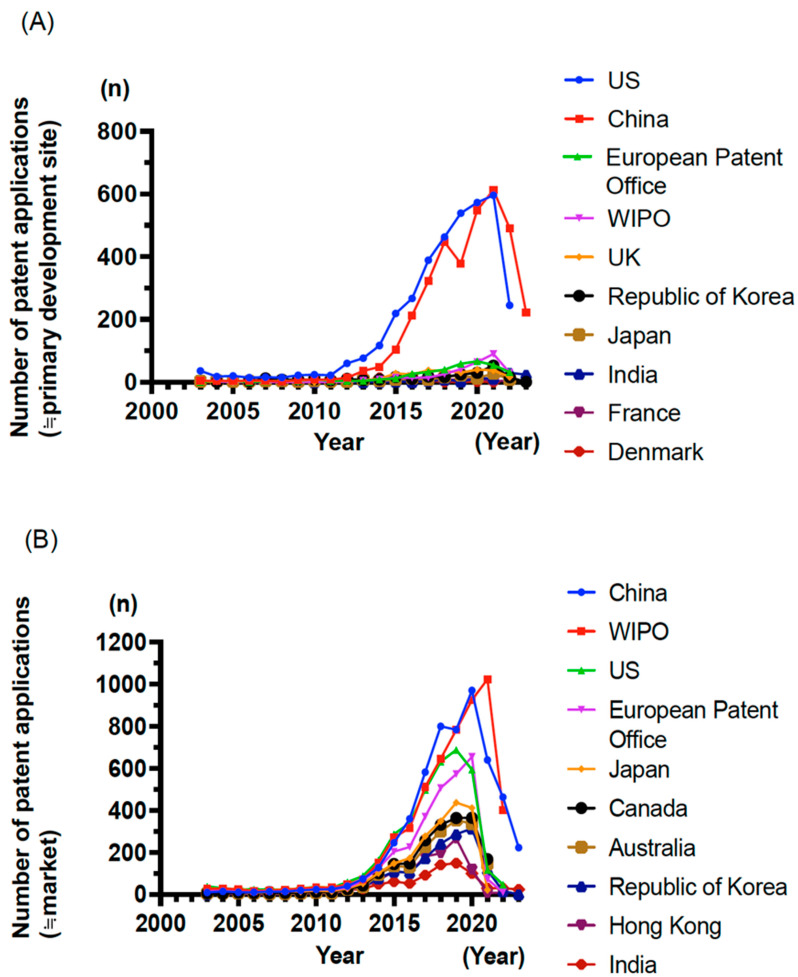
(**A**). Priority filing countries for CAR-T and TCR-T cell therapies. Countries where patent priority rights are claimed for CAR-T and TCR-T cell therapies are based on searches using a global patent database (Derwent Innovation-DWPI) and a pharmaceutical patent database (CCI-Patents). The vertical axis indicates the number of patent applications (≒primary development site), and the horizontal axis represents the year. The data from 2021–2023 are provisional and presented as reference values. (**B**). Most frequently designated countries for patent filings and the number of patents in CAR-T and TCR-T cell therapies. Countries most frequently designated as filing destinations for CAR-T and TCR-T cell-related patents are identified through the Derwent Innovation-DWPI and CCI-Patents databases. The vertical axis indicates the number of patent applications (≒market), and the horizontal axis represents the year. The data from 2021–2023 are provisional and presented as reference values.

An analysis of priority-filing countries (≒primary development site) revealed that the US and China dominate CAR-T/TCR-T-related patent applications (Figure 2A), indicating their central role in driving innovation. The European Patent Office ranked third, followed by the World Intellectual Property Organization (WIPO), the UK, Republic of Korea, and Japan. Among priority countries, the WIPO has emerged as the frequent destination (≒market) for patent filings (Figure 2B), suggesting a strong intent to pursue global protection. However, the filing patterns differ by country. While more than 50% of applications filed via the WIPO are from the US, Europe, Japan, and Republic of Korea, the corresponding proportions for China and India are lower at 20.9% and 16.7%, respectively (Table 3). These data suggest that although most Western countries aim for international commercialization, China and India prioritize domestic protection and deployment.

### 5.3. Top Assignees and Research Sectors

Among the top 20 patent assignees in the CAR-T/TCR-T domain, 11 are academic institutions (including universities, hospitals, and government/research institutes), and 9 are pharmaceutical or biotechnology companies (Table 4). This distribution reflects the translational nature of the field, in which academic discovery is rapidly moving toward clinical implementation and commercialization.

### 5.4. Technological Hotspots and Emerging Themes

The most frequently patented technological areas include immune cell engineering (e.g., T cells, NK cells, and stem cells), CAR structural components (e.g., intracellular signaling domains, transmembrane and extracellular domains, single-domain antibodies, and amino acid sequences), capable of binding antibody, immune response, and gene transfer technologies (e.g., lentiviral vectors and circular RNAs). These trends underscore the diverse innovations in the CAR-T/TCR-T cell therapeutic field.

### 5.5. Disease Focus and Clinical Translation Potential

Oncology remains the dominant therapeutic area in CAR-T and TCR-T intellectual properties. Initially, most filings focused on hematologic malignancies, such as leukemia, lymphoma, and multiple myeloma. In recent years, research and development of CAR-T cell therapy have been increasingly conducted for various solid tumors, including breast, ovarian, lung, prostate, liver, kidney, and pancreatic cancers. These indications remain largely experimental owing to challenges such as antigen specificity and tumor penetration; however, they represent high-priority targets for future expansion. Beyond specific diseases, active patenting is also observed in broader categories, such as adoptive cell therapies, immunotherapy and immune modulation, gene delivery vectors, effector and antibody fragments. These trends reflect the multidisciplinary nature of the field, which includes immunology, cell biology, genetic engineering, and biomanufacturing.

## 6. Conclusions and Future Directions

Despite significant scientific and clinical advances, the development of CAR-T and TCR-T cell therapies for solid tumors remains a formidable challenge. These modalities face multifaceted barriers, including antigen heterogeneity, an immunosuppressive TME, and complex manufacturing and delivery logistics. Nevertheless, the number of global clinical trials targeting solid tumors has markedly increased in the past decade, reflecting the growing momentum and investment in this field. Encouraging progress has been made in technological innovation and translational research. TCR-T cell therapy has achieved a historic milestone with the FDA approval of afamitresgene autoleucel for unresectable or metastatic synovial sarcoma. Although no CAR-T product has been approved for solid tumors, advances in engineering strategies, including in vivo programming, armored and dual-target CAR designs, and integration with checkpoint blockade, are rapidly pushing the boundaries of clinically achievable.

Moreover, insights from global patent activity indicate a vibrant and expanding ecosystem of innovation, driven by both academic institutions and biotechnology companies. The emergence of novel platforms such as mRNA-based or iPSC-derived CAR-T cell therapies points toward a future in which scalable, off-the-shelf, and safer cell therapies may become a clinical reality. Overcoming the unique challenges in solid tumors requires a synergistic approach that combines deep biological understanding, precise genetic engineering, and strategic clinical trial design. The next generation of adoptive cell therapies is likely to be characterized by enhanced specificity, persistence, safety, and accessibility.

In conclusion, although substantial hurdles remain, the collective efforts of global research and clinical communities are paving the way for the successful implementation of CAR-T and TCR-T cell therapies for solid tumors. Continued innovation with thoughtful integration into multidisciplinary cancer care holds great promise for transforming the treatment landscape of refractory solid tumors.

## Figures and Tables

**Table 1:** Comparison between CAR-T and TCR-T cell Therapies.

Feature	CAR-T Cell Therapy	TCR-T Cell Therapy
Target antigens	Surface antigens (e.g., CD19, BCMA)	Intracellular peptide antigens presented on MHC (e.g., NY-ESO-1, MAGE-A4)
T-cell receptor structure	CAR	TCR
Major histocompatibility complex (MHC) dependency	MHC-independent	MHC-dependent
Approved therapies (mainly for hematologic malignancies)	Tisagenlecleucel	Afamitresgene autoleucel
	Axicabtagene ciloleucel	
	Brexucabtagene autoleucel	
	Idecabtagene vicleucel	
	Lisocabtagene maraleucel	
	Ciltacabtagene autoleucel	

**Table 2:** Comparison of clinical and logistic characteristics between ex vivo and in vivo CAR-T cell manufacturing modalities.

Feature	Ex Vivo CAR-T Cell Therapy	In Vivo CAR-T Cell Therapy
Manufacturing process	T cells are collected from the patient, genetically engineered and expanded, then reinfused	Genetic material encoding the CAR is directly delivered into the patient’s T cells, typically via viral or non-viral vectors
Time to treatment	Prolonged, requiring several weeks due to leukapheresis, gene modification, expansion, and quality control	Potentially rapid if successful gene transfer occurs, allowing near-immediate therapeutic action
Cost	High owing to labor-intensive processes, facility requirements, and quality assurance testing	Low, as it may bypass complex manufacturing and reduce associated infrastructure needs
Feasibility	Clinically established in hematologic malignancies with multiple approved products	Currently under investigation in clinical trials

**Table 3:** Correlation between priority filing countries and designated countries. Correlation between priority filing countries (columns) and designated countries as patent jurisdictions (row) for CAR-T and TCR-T technologies, as analyzed using Derwent Innovation-DWPI and CCI-Patents. The values are shown as percentages.

	China	WIPO	US	European Patent Office	Japan	Canada	Australia	Republic of Korea	Hong Kong	India	Total (%)
US	38.3	88	-	51.9	36.1	39.7	35.5	25.3	21.8	13.4	100
China	-	20.9	7.1	6.5	4.7	2.2	2.5	3.2	2.9	0.7	100
European Patent Office	42.4	90.5	61.3	-	41.4	36.3	26.5	22.5	15.4	14.3	100
WIPO	44.3	-	43.1	35.2	26.7	22.3	23.6	18.2	13.5	10.4	100
UK	46.2	98.3	66.9	71	44.1	45.2	42.1	24.5	27.9	19.3	100
Republic of Korea	21	51.9	22.1	19.8	14.5	10.7	10.3	-	3.4	5	100
Japan	35.4	71.3	46.3	40.9	-	17.7	15.9	18.3	8.5	9.8	100
India	7.8	16.7	11.8	10.8	2.9	2	2	2.9	N/A	-	100
France	45.7	71.7	47.8	58.7	41.3	13	15.2	30.4	8.7	15.2	100
Denmark	39	100	92.7	92.7	70.7	73.2	75.6	39	12.2	36.6	100

**Table 4:** Top patent assignees in the CAR-T and TCR-T field. Leading patent assignees in CAR-T and TCR-T cell therapy-related filings, as determined through the analysis of Derwent Innovation-DWPI and CCI-Patents data. The columns of this table represent the major applicants of CAR-T/TCR-T related patents, and the rows indicate the year. The data from 2021–2022 are provisional and presented as reference values.

Assignee Type	Assignee Name	2003	2004	2005	2006	2007	2008	2009	2010	2011	2012	2013	2014	2015	2016	2017	2018	2019	2020	2021	2022	Total
Academia	University of Pennsylvania							1	1	5	18	10	24	19	11	17	30	22	9	25	4	196
Academia	University of California	1						1		2		3	2	8	5	4	13	37	23	25	12	136
Drug industry	Bristol-Myers Squibb Company							1	1			1	5	11	13	28	17	12	16	10	7	122
Drug industry	Novartis AG			1	2							9	20	13	11	13	19	7	7	3		105
Research institute	Memorial Sloan Kettering Cancer Center								1		1	3	6	9	10	12	15	11	10	20	2	100
Academia	University of Texas System						1		3	1		5	8	3	3	4	9	17	15	24	6	99
Drug industry	Autolus Therapeutics plc											5	5	11	8	15	17	15	10	4	3	93
Government	U.S. Department of Health and Human Services		1	1	1			2	1	3	3	5	6	9	6	13	11	8	8	11	4	93
Biotech	Shenzhen Binde Biotechnology														1	31	31	14	2	7	1	87
Hospital	City of Hope National Medical Center	8		3			1					1	4	5	7	5	6	10	13	9	2	74
Drug industry	Gilead Sciences, Inc.													2	10	12	14	5	8	11	4	66
Biotech	Miltenyi Biotec B.V. & CO. KG												3	4	9	11	11	8	11	2	6	65
Research institute	H. Lee Moffitt Cancer Center & Research Institute												1		3	7	14	11	11	11	7	65
Drug industry	Cellectis S.A.										7	7	15	9	7	10	2	1	3	2		63
Research institute	Institut national de la santé et de la recherche médicale				1							2	1	4	1	6	5	13	9	12	8	62
Academia	Stanford University		1											1	7	7	8	11	15	8	4	62
Hospital	The General Hospital Corporation			1								2		2	4	15	9	7	1	8	7	56
Drug industry	Legend Biotech Corporation													2	4	2	7	8	18	14	1	56
Biotech	CRISPR Therapeutics AG														1	16	6	8	12	9	3	55
Research institute	Fred Hutchinson Cancer Center								1	1	1	4	2	1	4	12	9	7	7	2	3	54

## Data Availability

Dataset available on appropriate and reasonable request from the authors.

## Abbreviations

The following abbreviations are used in this manuscript:

CAR	chimeric antigen receptor
FDA	Food and Drug Administration
GMP	good manufacturing practice
HLA	human leukocyte antigen
IL	interleukin
iPSC	induced pluripotent stem cell
MHC	major histocompatibility complex
NK	natural killer
TCR	T-cell receptor
TME	tumor microenvironment
WIPO	World Intellectual Property Organization
